# *Ganoderma lucidum Rhodiola* compound preparation prevent d-galactose-induced immune impairment and oxidative stress in aging rat model

**DOI:** 10.1038/s41598-020-76249-1

**Published:** 2020-11-06

**Authors:** Shuo Yuan, Yong Yang, Jiao Li, Xiaoyu Tan, Yuying Cao, Shaoheng Li, Hee-Do Hong, Liping Liu, Qinggao Zhang

**Affiliations:** 1 Chronic Diseases Research Center, Dalian University College of Medicine, Dalian, 116622 Liaoning China; 2 Department of Immunology and Pathogenic Biology, Yanbian University College of Basic Medicine, Yanji, 133022 Jilin China; 3 Xinhua Hospital Affiliated of Dalian University, Dalian, 116021 Liaoning China; 4 Korea Food Research Institute, Sungnam-si, South Korea

**Keywords:** Cytokines, Medical research

## Abstract

Aging is an irreversible process. This research aims to study the anti-aging effects of GRCP, a compound preparation made by *Ganoderma lucidum* and *Rhodiola rosen*, in aging rats. Rats were subcutaneously injected with 400 mg/kg of d-galactose daily, and aging could be induced after 8 weeks. The aging rats were treated with GRCP. This experiment was divided into 6 groups. Rats were randomly divided into the model group, positive control group, low-dose GRCP group (25 mg/kg body weight), medium-dose GRCP group (50 mg/kg body weight), and high-dose GRCP group (100 mg/kg body weight), healthy and normal rats were used as blank controls. After the end, the results show that the use of GRCP at a dose of 100 mg/kg is the best treatment for improving aging rats. Rats gained weight, spleen and thymus indexes, and splenocyte proliferation improved, and inflammatory cytokine levels decreased. Besides, biochemical indicators show that GRCP can improve the antioxidant enzyme activity and reduce the content of lipofuscin and TGF-β in aging rats (P < 0.05). GRCP can also inhibit the activation of the MyD88/NF-κB pathway in rat hippocampus. These results seem to suggest that GRCP can be used as a potential natural supplement or functional food to prevent aging.

## Introduction

Aging is a natural and inevitable process of organisms over time. It is a natural law in the course of human life and will cause the degradation of the body's tissues and organs^1^. It is estimated that by 2050, the global elderly population will reach 2.1 billion^2^. Therefore, delaying the aging process and improving the quality of healthy life in the elders have become one of the major problems in the medical community^3^. As aging is a series of body changes and dysfunctions caused by the interaction of multiple factors, in the past decades, it has been recognized that the aging process is associated with dysfunction of the antioxidant defense system, impaired immune system function, degraded cognitive and motor abilities^4^. Unbalanced metabolism of free radicals inside the body can destroy cell-related substances and cause the body to age. Besides, inflammatory factors are secreted during aging, preventing the immune system-mediated elimination of senescent cells^5^. Many works of literature have reported that antioxidants and immunostimulants are also beneficial for aging process^6–8^. Besides, with the continuous growth of age, the internal muscle and visceral tissue of the body gradually shrinks, the water content decreases, and the bone density decreases. Therefore, the body's weight decreases after aging. So, bodyweight is one of the commonly used indicators to assess aging. The use of Chinese medicine for anti-aging has a long history in China, and ancient medical scientists have created unique theories. Traditional Chinese medicine is widely classified, it has unique treatment methods. Many traditional Chinese medicine monomers have obvious anti-aging effects. They are suitable for conditioning chronic diseases, with fewer side effects and higher safety. Monomers such as curcumin and hypericin have great potential to slow the aging process^9,10^. However, the acquisition of Chinese medicinal materials is not easy, the cost is high, and the steps are complicated. The traditional Chinese medicine compound preparation is easy to obtain, rich in various active ingredients, and it has the characteristics of acting on multiple targets.

*Ganoderma lucidum* is an important traditional Chinese medicine. It has the effects of relieving cough and relieving asthma. Studies have shown that *Ganoderma lucidum* has a variety of biologically active ingredients^11^. It also has various pharmacological functions such as regulating the immune system, anti-tumor, anti-oxidation, and anti-radiation damage^12–14^. *Rhodiola* is a perennial herb with a long history of medicinal use. It often grows in high-altitude and hypoxic areas and can grow in extremely harsh and variable natural environments^15^. It has effects such as anti-radiation, anti-hypoxia, blood glucose control and so on^16–18^. *Ganoderma lucidum* and *Rhodiola* have rich pharmacological effects. Previous data have shown that a compound preparation made of *Ganoderma lucidum* and *Rhodiola* (GRCP) can significantly increase the anti-fatigue function of mice and enhance the immune function of immunodeficient mice^19,20^. Currently, the effect of GRCP on d-galactose-induced aging rats is unclear. Therefore, in this study we used d-galactose to induce the aging rat model, GRCP was administered to aging rats at different doses to assess the effect of GRCP on aging rats in vivo. The goal was to evaluate GRCP’s ability to improve aging in rats and provide rehabilitation support for its aging process.

## Results

### The main ingredients and content of GRCP

GRCP and its component analysis were provided by the Korea Food Research Institute (Table 1). According to the provided formula, the animals can be administered by gavage at a certain concentration.

**Table 1:** The main ingredients and content of GRCP.

Chemical content	Chemical content (mg/g)	Chemical composition (%)
Neutral sugar (glucose)	577.2 ± 12.1	71.8 ± 0.87
Uronic acid (galacturonic acid)	204.6 ± 13.8	25.4 ± 0.89
Protein (BSA)	7.0 ± 0.3	0.9 ± 0.02
KDO-liked material (KDO)	6.7 ± 0.2	0.8 ± 0.01
Total phenol (gallic acid)	9.0 ± 0.4	1.12 ± 0.04
Total	839.8 ± 33.8	100

Percentage (%) in each dry material.

*KDO* 2-keto-3-deoxy-d-manno-octulosonic acid.

### GRCP can increase weight in aging rats

**Figure 1:**
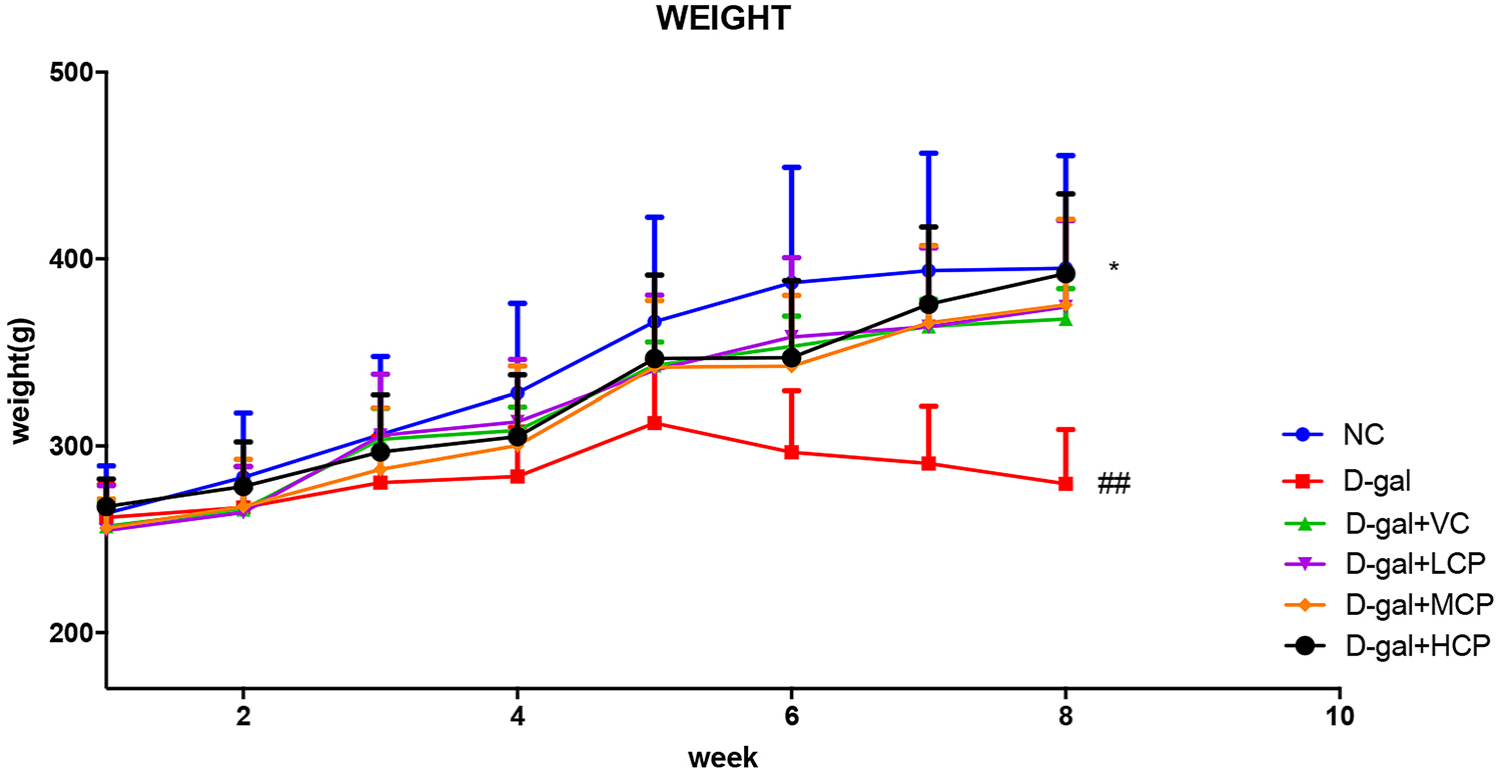
Results of body weight in aging rats. Results are expressed as mean ± SD (*n* = 8). ^##^P < 0.01 as compared with the NC group. *P < 0.05 as compared with the d-gal group.

Figure 1 shows the effect of GRCP on rat body weight over 8 weeks. The bodyweight of the d-gal group was reduced compared to that of the control group (P < 0.01). The body weight of rats in the d-gal + HCP group was higher than that in the d-gal group (P < 0.05). Compared with the d-gal group, rats in the LCP and MCP groups did not alter significantly body weight.

### Morris water maze test

**Figure 2:**
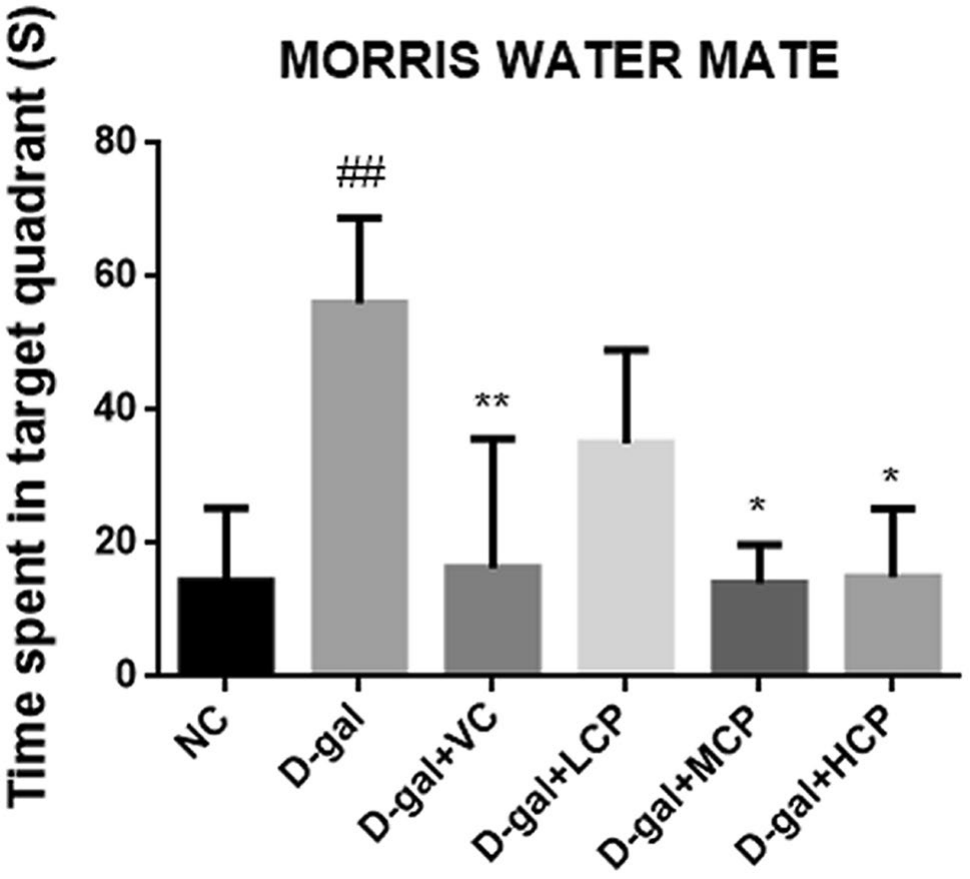
Results of the Morris water maze test in aging rats. Results are expressed as mean ± SD (*n* = 8). ^##^P < 0.01 as compared with the NC group. *P < 0.05 and **P < 0.01 as compared with the d-gal group.

Morris water maze test was performed to detect the effect of GRCP on spatial learning and memory ability. Figure 2 illustrates that compared with the NC group, rats in the d-gal group took a long time to find the hidden platform (P < 0.01). Compared with aging rats, the time required for rats in the VC group, MCP group, and HCP group was reduced (P < 0.05).

### GRCP affects the histopathology in aging rats

**Figure 3:**
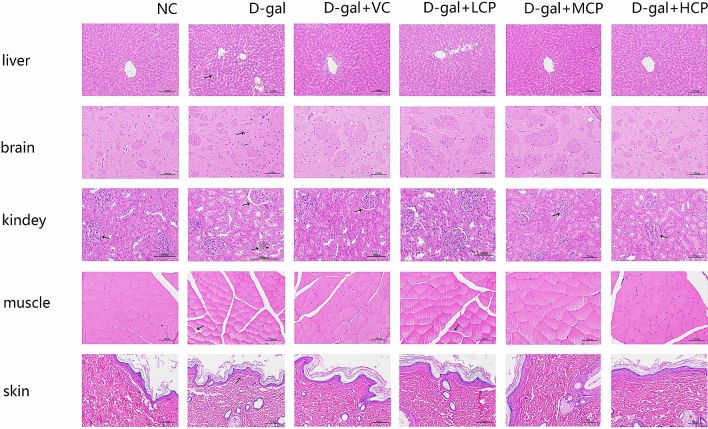
The effect of GRCP on histopathological changes of aging rats. The liver, brain, kidney, muscle, and skin were removed from rats of each group after GRCP treatment. Sections were processed for HE staining. The representative histopathological image at high magnification is shown (black bar, 100 μm).

To confirm whether GRCP has a protective effect on aging rats, the liver, brain, kidney, muscle, and skin tissues were detected. As shown in Fig. 3, the liver tissue of rats in the NC group showed no pathological changes, and the tissue color was relatively bright without dullness. The liver tissue of the d-Gal group had black streaks, and the cells became looser and the cell volume became smaller. However, the structure of hepatic lobules improved obviously, hepatocytes cord-like permutation was orderly and little lipofuscins deposited in GRCP administration groups.

In the NC group, the cell membrane of the brain cells of the rats was intact and continuous, and the cell morphology was intact, and the layers were compact. In the d-Gal group, the brain cells of the rats became smaller, the nucleus became condensed and fragmented, and local deep staining appeared. Compared with the d-Gal group, GRCP groups had complete cell morphology, clear cell edges, and uniform chromatin.

Compared with rats in the NC group, the rats in the d-gal group showed glomerular swelling, markedly increased volume and increased cells, and the gap with surrounding tissues became smaller. The symptoms of the drug group all improved.

The muscle fiber cells of the NC group rats were neatly arranged, polygonal, and the size was the same; the muscle fiber cells of the D-gal group showed degenerative changes, the muscle cells were rounded, and the intercellular space increased. In the GRCP treatment group, the close arrangement of muscle fibers was similar to those in the NC group.

The damage of the epidermal layer of the D-gal group rats increased, and the use of GRCP could reduce this change.

These results showed that GRCP could better protect D-gal induced liver, brain, kidney, muscle, skin damages in aging rats.

### GRCP can improve immune function in aging rats

**Figure 4:**
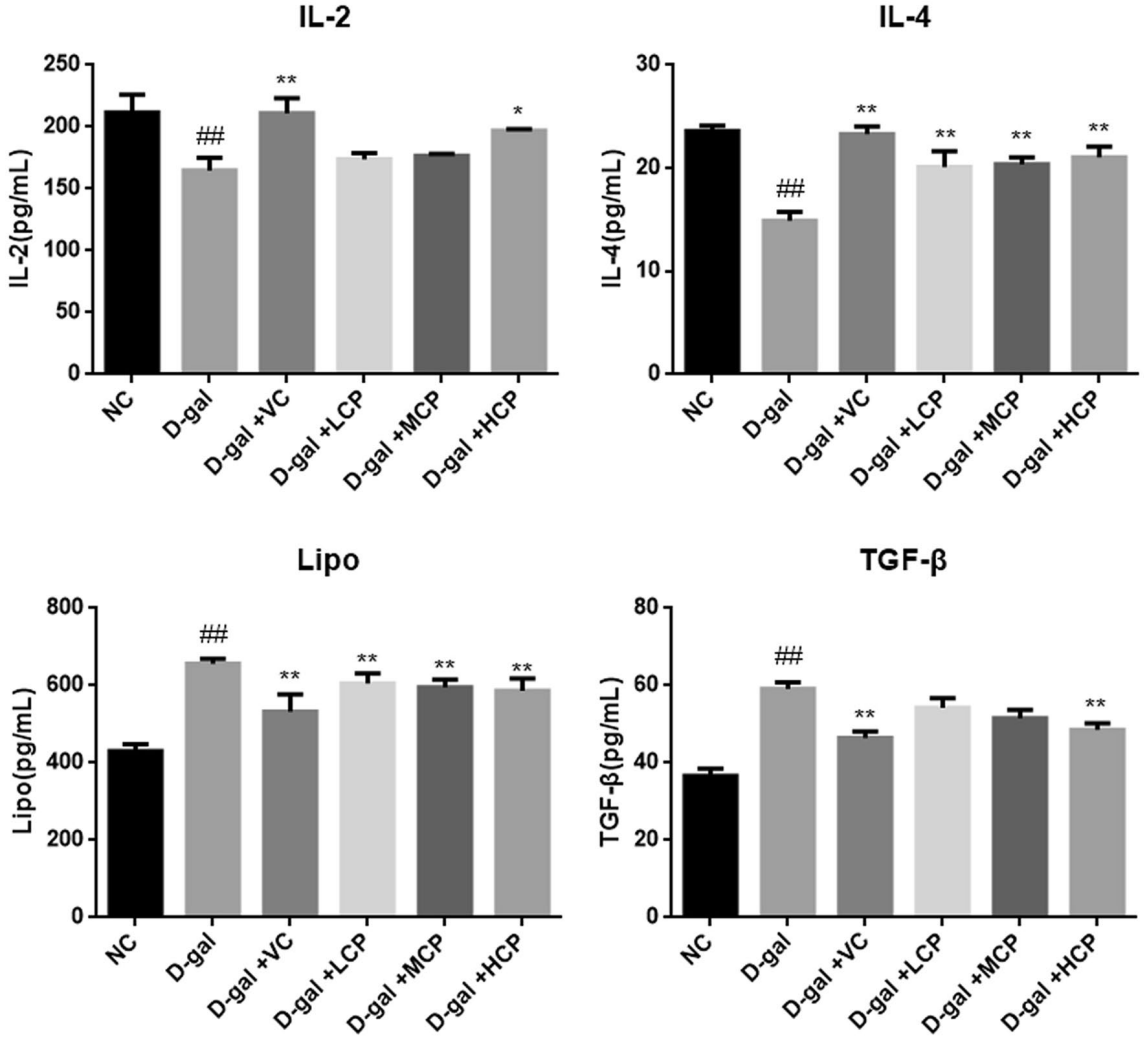
Results of immune function in aging rats. Results are expressed as mean ± SD (*n* = 8). ^##^P < 0.01 as compared with the NC group. *P < 0.05 and **P < 0.01 as compared with the d-gal group.

The thymus and spleen are important immune organs of the human body and are direct manifestations of immune function. As shown in Fig. 4, the spleen and thymus index were decreased in the D-gal group (P < 0.01). The spleen and thymus index were rebounded by VC and GRCP treatment, while 100 mg/kg GRCP was a superior treatment.

Besides, compared with the NC group, D-gal inhibited the proliferation of T and B lymphocytes of splenocytes (P < 0.01). The proliferative ability of T and B lymphocytes in the drug group was higher than that in the d-gal group. Furthermore, d-gal + HCP facilitated faster B and T lymphocyte proliferation of splenocyte (P < 0.01, P < 0.05).

### GRCP can reduce inflammatory factor levels in aging rats

**Figure 5:**
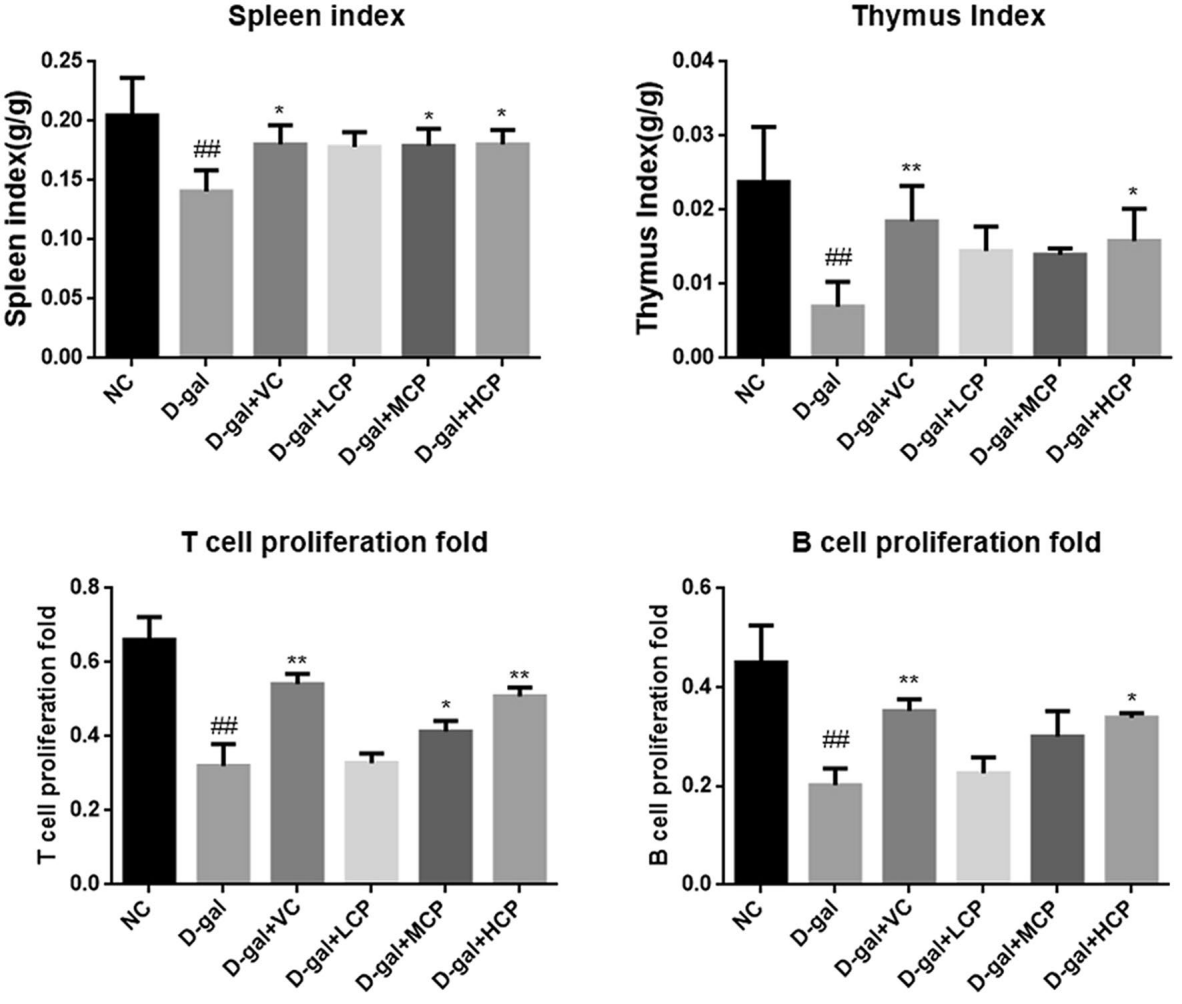
Results of IL-1β, IL-6, and TNF-α in aging rats. Results are expressed as mean ± SD (*n* = 8). ^#^P < 0.05 and ^##^P < 0.01 as compared with the NC group. *P < 0.05 and **P < 0.01 as compared with the d-gal group.

As shown in Fig. 5, compared with the NC group, the inflammatory factors IL-1β, IL-6, and TNF-α in the aging rats were significantly increased (P < 0.01). The VC group showed significant improvement (P < 0.01, P < 0.05). The inflammatory factors of the HCP group and MCP decreased significantly (P < 0.01, P < 0.05), and the level of IL-6 in the LCP group also decreased (P < 0.01).

### GRCP can increase IL-2 and IL-4 levels and reduce lipofuscin and TGF-β levels in aging rats

**Figure 6:**
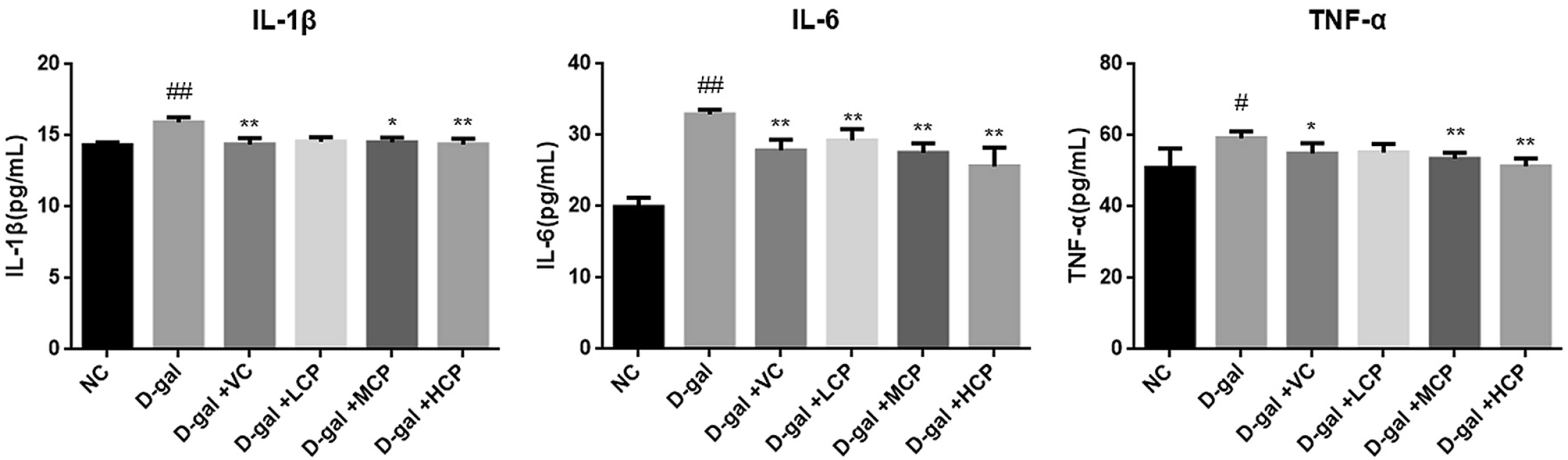
Results of IL-2, IL-4, Lipo, and TGF-β in aging rats. Results are expressed as mean ± SD (*n* = 8). ^##^P < 0.01 as compared with the NC group. *P < 0.05 and **P < 0.01 as compared with the d-gal group.

As shown in Fig. 6, compared with the NC group rats, the IL-2 level in the d-gal group was significantly reduced (P < 0.01), and the level of the rats in the VC group was significantly increased (P < 0.01). There were no significant statistics in the LCP and MCP groups rats. Rats in the HCP group had the most pronounced effect (P < 0.05). The level of IL-4 in the d-gal group rats was significantly reduced (P < 0.01), and the VC group, the LCP group, the MCP group, and the HCP group were significantly improved (P < 0.01).

Compared with the NC group, the Lipo level in the d-gal group was significantly increased (P < 0.01), and the VC group, LCP group, MCP group, and HCP group were significantly improved (P < 0.01). The TGF-β level in the d-gal group was significantly increased (P < 0.01), the level in the VC group was significantly reduced (P < 0.01), there was no significant statistical difference between the LCP and MCP groups, and the HCP group was significantly reduced (P < 0.01).

### GRCP can improve oxidative stress levels in aging rats

**Figure 7:**
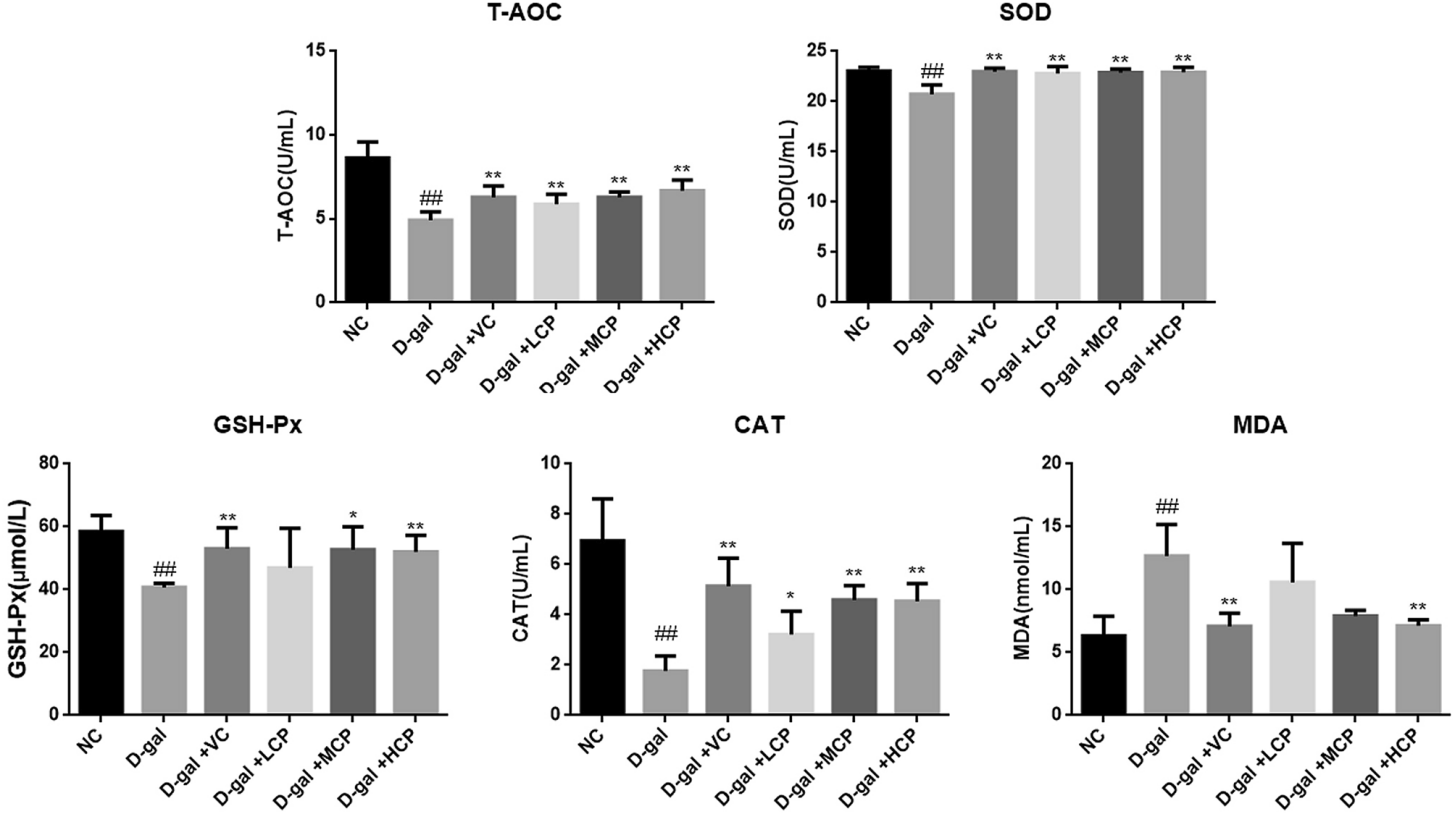
Results of the level of oxidative stress in aging rats. Results are expressed as mean ± SD (*n* = 8). ^##^P < 0.01 as compared with the NC group. *P < 0.05 and **P < 0.01 as compared with the d-gal group.

Compared with the NC group (Fig. 7), the T-AOC, SOD, GSH-PX, and CAT activities in the serum of the d-gal group rats were significantly reduced (P < 0.01). Compared with the d-gal group, the VC group, MCP, and HCP group were significantly improved (P < 0.01, P < 0.05). The levels of T-AOC, SOD, and CAT in the LCP group rats were also significantly increased (P < 0.01, P < 0.05).

Compared with the NC group, the MDA activity in the serum of the d-gal group was significantly increased (P < 0.01). Compared with the d-gal group, the VC group and HCP group were significantly improved (P < 0.01).

### GRCP can affect MYD88 and NF-κB protein levels in rat hippocampus

**Figure 8:**
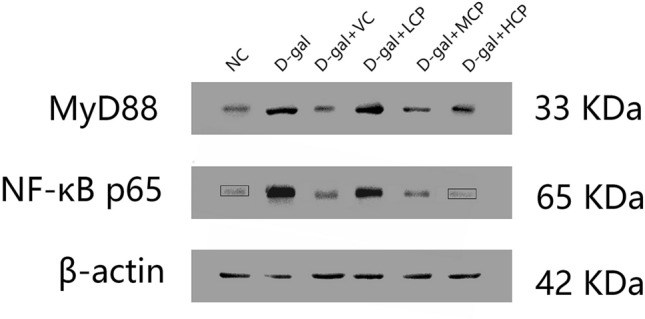
Results of MyD88/NF-κB in aging rats. Results are expressed as a mean ± SD (*n* = 8). ^##^P < 0.01 as compared with the NC group. *P < 0.05 as compared with the d-gal group.

The MYD88/NF-κB pathway plays a key role in the inflammatory response. We isolated rat hippocampus and performed Western-blot testing. As shown in Fig. 8, the expression of MyD88 and NF-κB p65 in hippocampus were significantly increased in d-gal group, compared to the NC group (P < 0.01). Moreover, a regulatory role of GRCP treatment on MyD88 and NF-κB expression in rat hippocampus was also observed. VC and GRCP administration reduced MyD88 and NF-κB p65 levels, while MyD88 and NF-κB p65 expression were also decreased dramatically in the HCP group (P < 0.05).

## Discussion

d-Gal actose is a monosaccharide. Excessive d-gal can lead to the accumulation of galactitol in the body, increase the type and number of ROS, accelerate organ aging, shorten the life of the body, cause behavioral disorders, and cognitive decline^21^. d-Gal is easy to obtain, simple to apply, and it has a low chance of inducing cancer. Besides, during the test of using d-gal, the experimental animal has a high survival rate. So, d-gal has been widely used in anti-aging pharmacological research^22^. In this experiment, we successfully induced the aging rats model by using d-gal, and no rat died during the modeling process.

Vitamin C is an essential nutrient for the human body. It is an effective antioxidant in the body. It can effectively remove free radicals and protect the body’s DNA, proteins, and lipids from oxidative stress. Vitamin C can be present inside and outside the cell after being absorbed by the body and plays a maintenance role in the damage caused by free radicals. Vitamin C can neutralize free radicals in the body, prevent macrophages from secreting superoxide radicals, and repair plasma membrane damage caused by oxidative stress through glutathione peroxidase and other pathways^23^. Therefore, in this experiment, we chose vitamin C as a positive control drug to evaluate the anti-aging effect of GRCP.

Bodyweight is one of the commonly used indicators for evaluating d-gal-induced aging models. The main purpose of the Morris water maze experiment is to evaluate the cognitive function and spatial memory function of rats. A study by Lai showed that the use of *Ganoderma lucidum* extract can increase the weight of aging rats^24^. Our experimental results show that normal rats have significant weight gain and d-galactose can affect normal physiological functions of rats. GRCP can increase body weight in aging rats. The results of the Morris water maze experiment show that it takes a long time for aging rats to find the platform. Other mice took less time than aging rats. Especially the HCP group performed best. This shows that GRCP not only has no side effects that affect the weight of rats, but that it can improve the physiological functions of aging rats. In addition, GRCP has the effect of improving learning and memory, interfering with cognitive dysfunction caused by d-galactose, and slowing down the behavioral problems caused by aging.

The liver, brain, kidneys, muscles, and skins are important organs and tissues in the body. With the increase of age, the structure of these organs and tissues will atrophy and damage, resulting in their functions gradually decline. In this study, the improvement of organ and tissue damage after GRCP treatment was shown. These data suggest that GRCP may be an effective method to exert its anti-aging effects in aging models.

Stress defense plays a very important role in the aging process, and resistance to oxidative stress may be an effective method to delay aging and treat aging-related diseases. Antioxidants in the body can remove various ROS and protect the body from damage caused by oxidative stress. T-AOC can reflect the total antioxidant capacity of the body. SOD is a natural free radical scavenger in the body and the first line of defense against oxidative damage. GSH-Px plays an active defense role in protecting the body from the damage of lipid peroxides, can protect the activity of hemoglobin and erythrocyte enzymes, and prevent oxidative damage to biological cell membranes. CAT can catalyze the decomposition of hydrogen peroxide that penetrates the cell membrane into oxygen and water, which is of great significance to the body's growth and development and metabolic activities. Excessive ROS will produce MDA, cause cross-linking and polymerization of large molecular substances such as proteins and nucleic acids, and have certain cytotoxicity^25–27^. Liu K showed that subcutaneous injection of d-gal can significantly reduce SOD, CAT, and GSH-Px levels and increase MDA levels in serum and skin tissues of mice. However, the use of high-dose *Ganoderma lucidum* protein can significantly increase the content of antioxidants, thereby reducing the content of MDA^28^. In addition, Maria M. found that *Rhodiola rosea* water extract can prolong the time life of yeast and increase cell SOD levels^29^. The results of this article show that T-AOC capacity, SOD content, GSH-Px content, and CAT content decrease in d-galactose-induced aging rats, while MDA content increases. This shows that the oxidative stress of aging rats is significantly enhanced. After GRCP intervention, the T-AOC capacity, SOD content, GSH-Px content, and CAT content in aging rats were increased to varying degrees, and the MDA content was also reduced to a certain extent. Especially the HCP group had the best effect. It shows that GRCP can improve the ability to scavenge oxygen free radicals in aging rats, reduce the damage of peroxidation products to the body, and then reduce the degree of aging in rats, and play an anti-aging effect.

During the aging process, the body’s internal immune system is fully involved in the entire aging process, and immune tissues and organs are affected to some extent. The thymus index and spleen index are closely related to the aging^30^. The thymus index and spleen index of aging rats were significantly decreased, and the proliferation of T cells and B cells was significantly reduced. After GRCP intervention, the thymus index and spleen index were improved to varying degrees, and the proliferation of T and B cells were improved. Rats in the HCP group had the best effect. This indicates that GRCP can enhance the immune function of cells in immunocompromised rats, not only T cell-mediated cellular immunity, but also B cell-mediated humoral immunity. Inflammatory cytokines are related to inflammation, including IL-1β, IL-6, and TNF-α, which can be rapidly released under pathological conditions, leading to an inflammatory response in the body^31,32^. The experimental results show that the level of inflammatory cytokines in aging rats is significantly increased, indicating that the immune function of the body is affected after aging, and the release of inflammatory factors is increased. After treatment with GRCP, the levels of IL-1β, IL-6, and TNF-α were significantly reduced. The above data shows that GRCP can inhibit the release of inflammatory factors during aging, thereby reducing the damage caused by inflammatory factors to the body by enhancing the body's immune function. In addition, we observed that GRCP also reduced lipofuscin, TGF-β levels, and increased IL-2 and IL-4 levels in aging rats. IL-2 can regulate the proliferation and apoptosis of activated T cells, and can also promote the production of immunoglobulins by B cells to exert anti-inflammatory effects. IL-4 is derived from the immune system that enhances the immune response to antigens. TGF-β is a pleiotropic cytokine, which is related to inflammation and metabolism. The accumulation of lipofuscin is related to oxidative stress, and lipofuscin can also promote the activation of macrophages, leading to chronic inflammatory processes^33–36^. From the above results, we conclude that GRCP can enhance the immune function of aging rats induced by d-gal actose to a certain extent.

Myeloid Differentiation Factor 88 (MyD88) plays an important role in mammalian host defense. MyD88 is a cytoplasmic soluble protein composed of two special domains that can form an inflammatory response pathway with Nuclear Factor Kappa-B (NF-κB), thereby inducing the release of inflammatory factors and amplifying inflammatory signals^37–39^. The transcription factor NF-κB is the main mediator of inflammation, usually composed of p50 and p65 proteins as components of inactive cytoplasmic complexes, and is combined with IκB, a member of the κB inhibitor family. When stimulated, IκB undergoes phosphorylation and targeted degradation, eventually leading to the transfer of NF-κB family members to the nucleus and a series of reactions that induce the release of inflammatory factors IL-1, IL-6, and TNF-α, amplifying inflammatory signals plays a key role in the body's immune function^40^. We took out the hippocampus of the rats after the modeling and extracted the protein for Western Blot experiments. The experimental results showed that the levels of MyD88 and NF-κB p65 in the hippocampus of aging rats were greatly increased. After treatment with GRCP, the contents of MyD88 and NF-κB decreased. Especially the effect of the HCP group was the most obvious. It was concluded that GRCP may inhibit the NF-κB signaling pathway by down-regulating the content of MyD88 in aging rats, while preventing the activation of NF-κB, inhibiting the secretion of inflammatory mediators, and achieving anti-aging effects.

In addition, GRCP contains nutrients such as neutral sugar, uronic acid, and protein, which has obvious anti-aging effects. The experimental results show that the effect of the HCP group is the most obvious.

In summary, d-gal -induced aging model rats have cognitive dysfunction, low immune function, and severe oxidative stress. The administration of GRCP can significantly improve the cognitive function of d-gal -induced aging model rats, improve the oxidative stress response of aging rats, enhance the immune function of aging rats, and reduce the secretion of inflammatory factors in aging rats. Its mechanism of action may be to block the NF-κB signaling pathway by reducing the MyD88 protein content in rats. GRCP is rich in nutrition and has obvious anti-aging effects. The rats in the HCP group are the most effective. The above experimental data provide a theoretical basis for further development of health food. This discovery laid the foundation for exploring the relevant mechanisms of *Ganoderma lucidum* and *Rhodiola* to delay aging and provided a new direction for future research.

## Materials and methods

### Materials and chemicals

GRCP was provided by Korea Food Research Institute. Fetal bovine serum (FBS), lipopolysaccharide (LPS), concanavalin (ConA), and methyl phenazine sulfate (PMS) were purchased from Sigma-Aldrich Co. (St. Louis, Mo, USA). Methyl trichlorosilane (MTS) was purchased from American Promega Co. RPMI Medium 1640 basic was purchased from Thermo Fisher Scientific (China) Co. Ltd. Protein extraction kit, Vitamin C (VC), d-galactose, and Red Blood Cell Lysis Buffer were bought from Beijing Solarbio Technology Co. Ltd. The assay kits for Total antioxidant capacity(T-AOC), Superoxide Dismutase (SOD), glutathione peroxidase (GSH-Px), Catalase (CAT), and malondialdehyde (MDA) were the products of Nanjing Jiancheng Bioengineering Institute (Nanjing, China). The enzyme-linked immunosorbent assay (ELISA) kit of Interleukin 1β (IL-1β), Interleukin 2 (IL-2), Interleukin 4 (IL-4), Interleukin 6 (IL-6), lipofuscin (Lipo), Tumor necrosis factor α (TNF-α) and Transforming Growth factor β(TGF-β) were purchased from Shanghai Yuchun Bio-tech Inc (Shanghai, China). All the other chemicals and reagents used in this study were of analytical grade made in China unless otherwise indicated. The experimental water was distilled water prepared by our laboratory.

### Preparation of the GRCP

The powdered drug of GRCP was dissolved in deionized water to prepare three different concentrations of high (100 mg/kg body weight), medium (50 mg/kg body weight), and low (25 mg/kg body weight).

### Animal model and treatment

Male SD rats (2 months old, weighing 180 ± 20 g) were obtained from the Animal Facility of Dalian Medical University. The animals were raised under conditions at 23 ± 2 °C with a relative humidity of 55 ± 10%. The health of the rats was monitored every day, and they had no stress or discomfort in the cage. Regulations of Experimental Animal Administration issued by the State Committee of Science and Technology of China, and all the experimental protocols and experimental procedures were approved by the Ethics Committee for Animal Experimentation of Dalian University of Medicine (Dalian, China). A total of 48 rats were randomly assigned to six groups of 8 rats per group. Group 1 (Normal control group, NC): normal rats were injected with the same volume of physiological saline and administrated intragastrically with distilled water solution. Group 2 (Model control group, d-gal): d-gal rats were injected subcutaneously with 400 mg/kg d-gal and administrated intragastrically with distilled water solution. Group 3 (Positive control group, d-gal + VC): d-gal + VC rats were injected subcutaneously with 400 mg/kg d-gal and administrated intragastrically with 100 mg/kg VC. Group 4 (d-gal and low dose of GRCP group, d-gal + LCP): d-gal + LCP rats were injected subcutaneously with 400 mg/kg d-gal and administrated intragastrically with 25 mg/kg GRCP. Group 5 (d-gal and middle dose of GRCP group, d-gal + MCP): d-gal + MCP rats were injected subcutaneously with 400 mg/kg d-gal and administrated intragastrically with 50 mg/kg GRCP. Group 6 (d-gal and high dose of GRCP group, d-gal + HCP): d-gal + HCP rats were injected subcutaneously with 400 mg/kg d-gal and administrated intragastrically with 100 mg/kg GRCP. Rats were injected once a day for 8 weeks, then they were sacrificed at the end of treatment, and serum, organs, and tissues were immediately collected for experiments.

### Histological examination

The tissue samples, such as liver, brain, kidney, muscle, and skin, were collected and fixed in formalin. Then the samples were dehydrated by immersion in xylene and embedded in paraffin. The tissue sections were dyed using hematoxylin and eosin (H&E) staining. Finally, the tissues from each group were examined under light microscopy.

### Determination of body weight, spleen index, and thymus index

During the treatment, the health status of the rats was observed every day. The bodyweight of the rats was measured once a week for analysis. Upon sacrifice, the thymus and spleen of the rat were separated in a sterile environment and weighed on an electronic tissue scale. The thymus index and spleen index were calculated according to the following formula: thymus (spleen) index (g/g) = thymus (spleen) mass (g)/weight (g).

### Splenocyte proliferation

In a sterile environment, place the spleen of the rat in a petri dish, cut the spleen with ophthalmic scissors, add 1 ml RPMT 1640 cell culture solution, gently grind the spleen with a 5 ml syringe core, and add 1 ml RPMT 1640. Spleen cells were filtered through a 200-mesh sieve, collected in a sterile centrifuge tube, and centrifuged at 1000 r/min and 4 °C for 5 min. Discard the supernatant and add 3 ml erythrocyte lysate. Resuspend, 4mins later, add 2 ml RPMT 1640 to resuspend and wash. Centrifuge, and repeat washing once. Then add 1 ml RPMT 1640 to resuspend and make a spleen cell suspension.

Splenocytes (3 × 10^6^/ml) were plated on 24-well plates. Add 1 ml splenocytes and concanavalin to T-cell proliferation wells, add 1 ml splenocytes and lipopolysaccharide to B-cell proliferation wells. The final mass concentration of concanavalin was 2 µg/ml, and the final mass concentration of lipopolysaccharide was 10 µg/ml. After 68 h of incubation in a 24-well plate, transfer them to a 96-well plate. Transfer 100 µl of each well, and add 40 µl/well of a mixture of MTS and PMS. The ratio of MTS and PMS is 20:1. After 5 h of incubation, the absorbance of each well was detected with a microplate reader, and the detection wavelength was 492 nm.

### Measurement of IL-1β, IL-2, IL-4, IL-6, Lipo, TNF-α, and TGF-β

According to the instruction manual, use ELISA kit to quantitatively detect the levels of IL-1β, IL-2, IL-4, IL-6, Lipo, TNF-α, and TGF-β in serum. The absorbance was measured at a wavelength of 450 nm.

### Determination of T-AOC, SOD, GSH-PX, CAT, and MDA

Blood serum was obtained for the measure. The levels of T-AOC, SOD, GSH-PX, CAT, and MDA were determined by kits. All procedures were performed according to the manufacturer’s instructions.

### Morris water maze

The swimming water maze test uses a circular pool (150 cm in diameter and 120 cm in height, filled with a depth of 70 cm in 22 ± 0.5 °C water). An escape platform about 1 cm below the water surface is placed in the pool and the camera is used to record the swimming activity overhead. The aged mice were trained for 4 days in advance and then started the formal experiment. Each mouse was trained twice a day, each time lasting 60 s or ended immediately when the mice jumped to the escape platform in the water. Detect the escape time, that is, the time it takes for each mouse to start from the water and jump to the escape platform.

### Western blot

The protein was extracted from rat hippocampus using a protein extraction kit, and electrophoresis was performed on a 10% SDS-PAGE gel. Added 60 μg protein to each well. The separated proteins were transferred to a polyvinylidene fluoride membrane. After blocking with 5% skim milk protein for 1 h at room temperature, the cells were treated with MyD88 (1: 2000; Elabscience, China), NF-κB p65 (1: 1000; CST, USA) and β-actin (1: 1000 Santa Cruz, USA) at 4 °C overnight. After washing, membranes were incubated with secondary antibody (1:10,000; Affinity, China) at room temperature for 1 h. After washing three times with TBST, the membrane was observed under a gel imager (BIO-RAD, USA), which used Tanon ECL high sensitivity chemiluminescence reagent (Tanon, China). β-actin was used as an internal control. The analysis was performed using Image Lab software.

### Animal statement

Animal experiments were performed following the approved international guide for the Care and Use of Laboratory Animals, including any relevant details. All experiments were approved by the Committee on the Ethics of Animal Experiments of the Dalian University, China.

## Data Availability

Data generated during the current study are available from the author upon request.
